# Do bioresorbable polyesters have antimicrobial properties?

**DOI:** 10.1007/s10856-017-6021-5

**Published:** 2018-01-16

**Authors:** Lukas Gritsch, Christopher Lovell, Wolfgang H. Goldmann, Aldo R. Boccaccini

**Affiliations:** 1 Institute of Biomaterials, University of Erlangen-Nuremberg, Cauerstraße 6, 91058 Erlangen, Germany; 2 Lucideon Ltd., Queens Road, Penkhull, Stoke-on-Trent, Staffordshire, ST4 7LQ UK; 3 Department of Biophysics, University of Erlangen-Nuremberg, Henkestrasse 91, 91052 Erlangen, Germany

## Abstract

**Abstract:**

Biodegradable and bioresorbable polyesters (BBPEs) are a widespread class of aliphatic polymers with a plethora of applications in the medical field. Some reports speculate that these polymers have intrinsic antibacterial activity as a consequence of their acidic degradation by-products. The release of organic acids as a result of the hydrolytic degradation of BBPEs in vivo and the resulting pH drop could be an effective inhibitor of the growth of pathogens in the local environment adjacent to BBPE-based devices. However, there is no clear and conclusive evidence in the literature concerning the antibacterial activity of BBPE to support or refute this hypothesis. In this communication we address this point through an assessment of the antibacterial properties of six well-established commercially available BBPEs. Agar diffusion assays and optical density measurements at 600 nm were performed on all the polymer samples to characterize the growth of bacteria and any potential inhibition over an incubation period of 24 h. The results indicated that BBPEs do not possess an intrinsic and immediate antibacterial activity, which is consistent with the clear mismatch between the time-scales for bacterial growth and the rate of degradation of the polyesters.

**Graphical abstract:**

## Introduction

Biodegradable and bioresorbable polyesters (BBPEs) possess numerous beneficial properties (e.g., tailorable mechanical properties, tunable degradation in contact with biological fluids, high availability at competitive costs, and drug carrying/delivering capability), which make them suitable for numerous medical applications such as surgical sutures, orthopedic clips, screws and staples, stents, tissue engineering scaffolds, coatings, and drug delivery vehicles [1, 2]. BBPEs degrade through a hydrolytic process to form by-products, which are metabolized by the body through physiological pathways [3, 4]. Since these by-products are acidic (e.g., lactic acid), it has been proposed that the degradation might result in a drop of the local pH below 4, thus inhibiting the growth of pathogens [2, 5, 6]. However, the literature lacks evidence that pH gradient dependent bacterial starvation actually happens in relation with the degradation of BBPEs. The statement that pH related antibacterial effects occur upon degradation of BBPEs is thus a hypothesis without a solid empirical validation.

In this short communication we present the results of two well-established protocols of bacterial inhibition assays performed on an array of commercially available polyesters (in film form) to assess the effect of the polymer degradation products on the growth of common strains of both Gram-positive and Gram-negative bacteria.

## Materials and methods

### Preparation of the films

Films of six different commercially available BBPEs were prepared by solvent casting. The polymers selected in this study are summarized in Table 1.

**Table 1:** Summary of the polymers used in this study

Sample	Polymer (full name)	Supplier	Molecular weight
P1	Poly (d,l-Lactide-co-glycolide) (PLGA)	Vornia Biomaterials, Ireland	5–15 kDa
P2	Poly (l-Lactide) (PLLA)	Vornia Biomaterials, Ireland	300–400 kDa
P3	Polylactic acid (PLA)	Goodfellow GmbH, Germany	N/A
P4	Polyhydroxybutyrate (PHB)	Goodfellow GmbH, Germany	N/A
P5	Poly (ε-caprolactone) (PCL)	Sigma-Aldrich, Germany	80 kDa
P6	Poly(d,l-Lactide) (PDLLA)	Corbion Purac, Netherlands	80 kDa

All polymers were dissolved in 20 mL of dichloromethane (DCM) at a fixed concentration of 5% w/v. The solutions were then poured into glass petri dishes and left to dry at room temperature for 48 h. After drying, disk shaped samples (films of *Ø* = 12 mm and 0.2 mm thickness) were cut from the as-cast film and then stored under vacuum to remove any residual solvent.

### Bacterial culture

#### Bacterial strains

Gram-positive *Staphylococcus carnosus* and Gram-negative *Escherichia coli* were chosen as model bacterial strains for the biological testing. Prior to the experiment, suspensions of the selected strains in nutrient broth were prepared as follows: a bacterial colony was suspended in 5 mL of lysogeny broth (LB broth #968.1, Carl Roth GmbH) and grown overnight in an orbital shaker at 37 °C. The obtained suspension was diluted to adjust its optical density to reach the value of 0.015 at OD 600 _nm_ (Biophotometer Plus, Eppendorf AG, Hamburg, Germany).

#### Halo tests

Antibacterial agar diffusion assays (halo tests) were carried out according to a previously developed protocol [7]. Briefly, 20 µL of the prepared bacterial suspension was deposited and spread homogeneously onto a petri dish of 10 cm diameter, which was previously covered with a uniform layer of fresh agar (LB Agar (Lennox), Lab M Ltd.). The polyester samples were then placed onto the agar and the culture was incubated for 24 h at 37 °C and high relative humidity (~80%). After the incubation time, the inhibition zone around each sample was assessed. High-resolution images of the agar plates were taken with a digital camera (Nikon D90).

#### Optical density

For turbidity measurements, samples of all types of polyester were immersed in 5 mL of the prepared bacterial suspension at an optical density of 0.015, measured with the same Biophotometer Plus, as previously described, and incubated in an orbital shaker at 37 °C. At given time-points (1, 3, 6, and 24 h) aliquots of the bacterial suspensions were withdrawn and the variations in optical density were analyzed.

## Results

### Halo tests

**Fig. 1:**
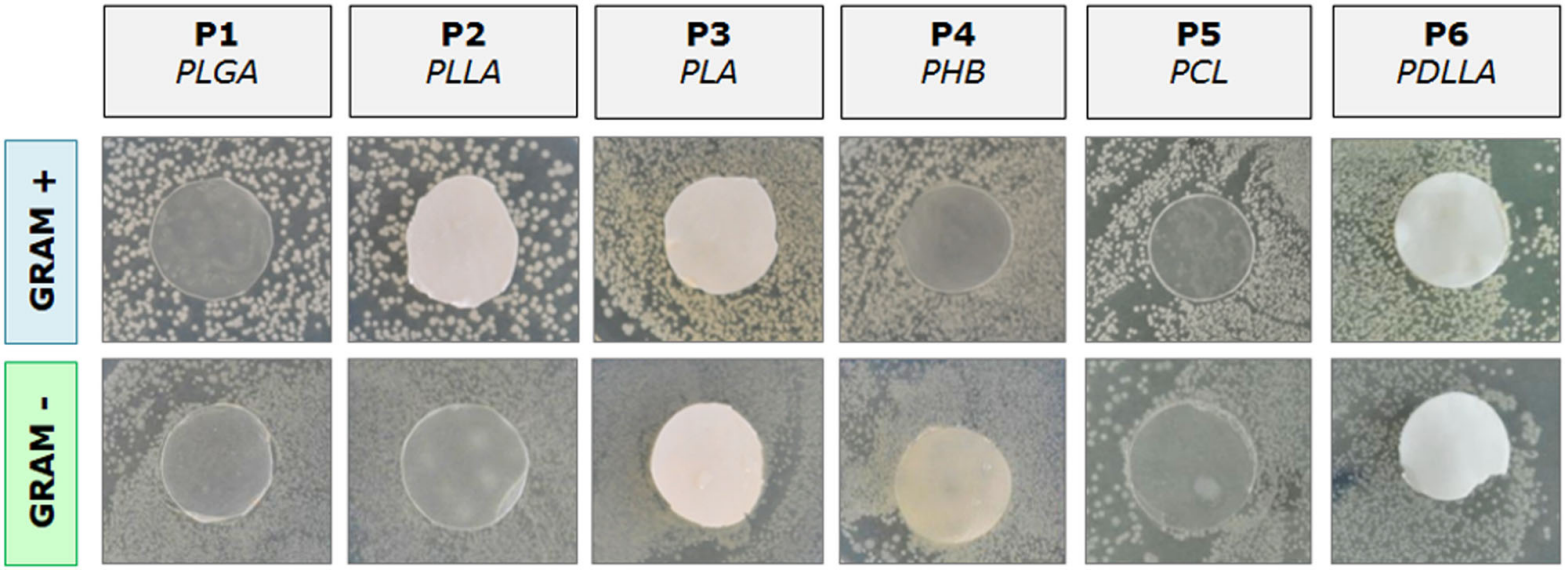
Area of inhibition of tested polyesters against both Gram + (*Staph. carnosus*) and Gram- (*E. coli*) bacteria after 24 h

After 24 h of incubation the polyesters showed no inhibition area, either with Gram-positive or –negative bacteria. The results of the halo tests were generally negative and bacteria grew without any visible alteration around the samples (Fig. 1). Based on these simple results, it is safe to conclude that the tested commercial polymers have no antimicrobial effect on the selected strains. Furthermore, it is can be stated that the absence of inhibition is due to the lack of change in pH around the sample.

### Optical density

**Fig. 2:**
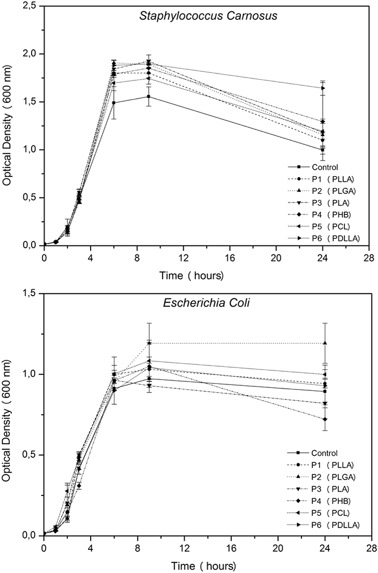
Turbidity measurements on suspensions of both tested strains in LB medium. All curves follow the standard development of unconditioned bacteria growth for the different BBPEs investigated

Similar results were obtained also from turbidity measurements, as shown in Fig. 2, confirming that all six polyesters have no significant effect on the growth of bacteria suspended in medium. All curves resemble a typical sequence of bacterial growth [8] and are comparable (*p* < 0.05) to the control. Measurements of the pH of the suspensions revealed that no pH drop occurred. This can be a consequence of both the buffer capability of the medium and the lack of any consistent degradation of the materials within the considered period of time.

## Discussion

It is well known that most common pathogens can grow effectively only within a pH window of 4 to 9 [9–12]. Moreover, organic acids have been proven to be an effective inhibitor of bacterial growth since they interfere with the normal functions of the bacterial membrane [11–13]. The internal pH of bacteria must remain close to neutral even when the external pH changes. Bacteria maintain homeostasis by means of ion pumps, however, if the shift from neutrality is too large, H^+^ ions start to migrate into the cytoplasm, acidifying it.This effect alters the ionization of biomolecules, inhibits the activity of enzymes and transmembrane proteins and disrupts the plasma membrane [8]. These phenomena act concurrently, gradually blocking the ability of correct cellular respiration of the microorganism. From the cell growth point of view, the pH-dependent bacterial inhibition will have an effect only at the level of maximum population, with no influence on the lag phase or the rate of growth [8].

Our results highlight that the mechanisms described above cannot take place because of the existence of a fundamental time mismatch between the degradation-driven pH drop and the growth of bacteria. While the former is a relatively slow process, occurring over periods of weeks to months, the latter happens within circa 1–2 days. As a consequence, the inhibition of bacteria caused by low pH described above cannot occur and the bacteria form a strong and resilient colony long before the materials evaluated herein start to degrade, negating de facto a possible pH-dependent antimicrobial effect. A potential way to overcome this discrepancy would be to have a fast degrading polymer that is able to acidify the pH of the biological environment within a few hours. However, in this case, there could be an increased risk of inflammation of the surrounding tissues. Indeed, previous reports on PLA-based orthopedic implants identify the local pH drop as one of the main causes of chronic inflammation and, eventually, of failure [14, 15]. These facts highlight the need to characterize in-depth the interaction between bacteria and degrading BBPEs to confirm whether the hypothesized antimicrobial activity of these polymers can be a promising strategy in fighting bacteria, which could see an expansion of the scope of medical applications of BBPEs.

## Conclusions

In order to test the hypothesis, often found in literature, that biodegradable bioresorbable polyesters can have a pH-dependent antimicrobial effect, an array of six commercially available BBPEs have been biologically characterized. Two well-established assays were performed on test strains of both Gram-positive and Gram-negative bacteria. No pH-dependent antimicrobial effect of any analyzed polyester (in film form) was detected. Since there is a strong rate difference between the fast growth of bacteria and the relatively slow degradation of the polymers, no inhibition can occur. Therefore, our results disprove that BBPEs are intrinsically antimicrobial.
